# When Cancer Mimics Pain: Maxillary Primary Intraosseous Carcinoma Misdiagnosed as Trigeminal Neuralgia

**DOI:** 10.3390/dj14010028

**Published:** 2026-01-04

**Authors:** Coșarcă Adina Simona, Száva Daniel, Gherman Mircea Bogdan, Mocanu Simona, Petrovan Cecilia, Mihai-Vlad Golu, Ormenişan Alina

**Affiliations:** 1 Department of Oral and Maxillofacial Surgery, George Emil Palade University of Medicine, Pharmacy, Science and Technology of Targu Mures, 540142 Targu Mures, Romania; adina.cosarca@umfst.ro (C.A.S.); daniel.szava@umfst.ro (S.D.); cecilia.petrovan@umfst.ro (P.C.); alina.ormenisan@umfst.ro (O.A.); 2 Department of Neurosurgery, Emergency County Hospital Târgu Mures, Gheorghe Marinesscu Street, nr 50, 540136 Targu Mures, Romania; 3 Department of Histology, Emergency County Hospital Târgu Mures, Gheorghe Marinesscu Street, nr 50, 540136 Targu Mures, Romania; slmocan70@gmail.com

**Keywords:** primary intraosseous carcinoma, odontogenic carcinoma, trigeminal neuralgia misdiagnosis, dural invasion, multidisciplinary management

## Abstract

**Background**: Primary intraosseous carcinoma (PIOC) is a rare and aggressive odontogenic malignancy that originates within the jaw bones without initial mucosal involvement. Its atypical and nonspecific symptoms frequently lead to diagnostic delays, especially in maxillary presentations. **Methods**: A 74-year-old male presented with persistent trigeminal-like neuralgic pain along the ophthalmic branch, initially misdiagnosed as secondary trigeminal neuralgia. MRI revealed a 45 × 46 × 34 mm mass occupying the right maxillary sinus with orbital wall destruction and dural invasion. Following histopathological confirmation of malignancy, a multidisciplinary team performed total maxillectomy with orbital exenteration and dural resection, followed by reconstruction using a temporoparietal flap. Adjuvant radiotherapy was administered. **Results:** Histopathology revealed invasive odontogenic carcinoma with atypical squamous features, dentinoid deposition, and perineural invasion. Postoperative recovery was uneventful, with complete pain resolution. MRI and PET surveillance over 2.5 years demonstrated no local recurrence. **Conclusions:** Maxillary PIOC may present exclusively with neuropathic pain, mimicking trigeminal neuralgia and leading to delayed diagnosis. In cases of unexplained facial pain with sinus or skull base involvement, odontogenic malignancies should be considered in the differential diagnosis. Early imaging and multidisciplinary management are key to achieving timely diagnosis, effective treatment, and improved quality of life.

## 1. Introduction

Primary intraosseous carcinoma, not otherwise specified (PIOC, NOS), is a rare malignant odontogenic tumor that arises centrally within the jaw bones without initial connection to the oral mucosa. It is believed to originate from odontogenic cyst epithelium, remnants of odontogenic epithelium, reduced enamel epithelium surrounding impacted teeth, or other benign odontogenic precursors [1].

Historically, multiple terms were used to describe this entity, including primary intraosseous squamous carcinoma, cytogenic type; solid-type primary intraosseous squamous carcinoma; and intra-alveolar epidermoid carcinoma. However, according to the latest WHO (World Health Organization) Classification of Head and Neck Tumors 2024 [1]. These terms are now obsolete, and the use of unified nomenclature is strongly recommended to avoid diagnostic confusion. PIOC is currently categorized under malignant odontogenic carcinomas, NOS type, which highlights its unique origin and biological behavior.

This tumor occurs more frequently in the mandible, particularly in the posterior region and ramus, and is less common in the maxilla. Establishing an intraosseous origin is essential for diagnosis, requiring the exclusion of secondary invasion from alveolar mucosal squamous cell carcinoma or maxillary sinus carcinoma. Early stage PIOC may be asymptomatic and incidentally discovered during routine imaging. In more advanced cases, clinical features include pain, swelling, facial or labial paresthesia, cortical perforation, tooth mobility, pathological fractures, and occasionally regional lymph node metastasis, reported in approximately 40% of cases [1].

Radiologically, the tumor typically presents as a poorly defined osteolytic lesion with or without root resorption and cortical erosion. In cases arising from odontogenic cysts, a multilocular or scalloped radiolucency may be seen, mimicking a benign lesion such as a residual cyst, radicular cyst, dentigerous cyst, or odontogenic keratocyst, contributing to diagnostic challenges and frequent misdiagnosis [2].

PIOC is exceptionally rare, with most reports limited to individual case reports or small case series. A 2020 systematic review documented only 257 cases in the literature, with most arising from odontogenic cysts, particularly residual and radicular cysts [3]. Although predominantly occurring in adults, pediatric cases have also been reported [4,5].

Histopathologically, PIOC is characterized by islands and nests of atypical squamous epithelium with minimal keratinization, prickle cells, and varying degrees of cytologic atypia. The majority are moderately differentiated, and necrosis may be present [6,7]. The overall prognosis remains poor, with reported two- and four-year survival rates of 60.5% and 39.9%, respectively [6].

In this report, we describe a rare maxillary PIOC with orbital and dural invasion, initially misdiagnosed as trigeminal neuralgia due to its atypical clinical presentation dominated by neuropathic facial pain. This case emphasizes the diagnostic pitfalls associated with PIOC involving the maxilla and highlights the importance of early imaging and multidisciplinary management to achieve optimal outcomes.

## 2. Case Report and Clinical Management

The patient was diagnosed and treated at the Department of Oral and Maxillofacial Surgery, Emergency County Hospital, Târgu Mureș, Romania. All relevant clinical, radiological, surgical, and histopathological data were retrieved from the institutional medical records, with written informed consent obtained from both the patient and his family. The study was conducted in accordance with the ethical principles of the Declaration of Helsinki and was approved by the Institutional Ethics Committee of Emergency County Hospital, Târgu Mureș, Romania (protocol code: Ad 29513; date of approval: 10 December 2025).

A 74-year-old male presented to the Department of Oral and Maxillofacial Surgery with severe unilateral facial pain along the trajectory of the ophthalmic branch (V1) of the trigeminal nerve, radiating to the right frontal region. The pain was described as sharp, stabbing, and paroxysmal, with nocturnal exacerbation, significantly impairing sleep and quality of life.

The patient’s medical history included a previous varicella zoster virus infection affecting the same nerve distribution, for which he had been evaluated by a neurologist one year earlier. No other relevant systemic or maxillofacial medical history was reported.

On clinical examination, no intraoral, facial, or neurological abnormalities were observed. There was no mucosal ulceration, facial swelling, tooth mobility, or sensory deficit. Based on the pain characteristics and previous history, a diagnosis of secondary trigeminal neuralgia (ophthalmic branch) was initially considered, and conservative management was initiated using nonsteroidal anti-inflammatory drugs (3×/day) and vitamin B-complex (2×/day). Despite treatment, symptoms progressively worsened, with persistent and intense nocturnal pain.

**Figure 1:**
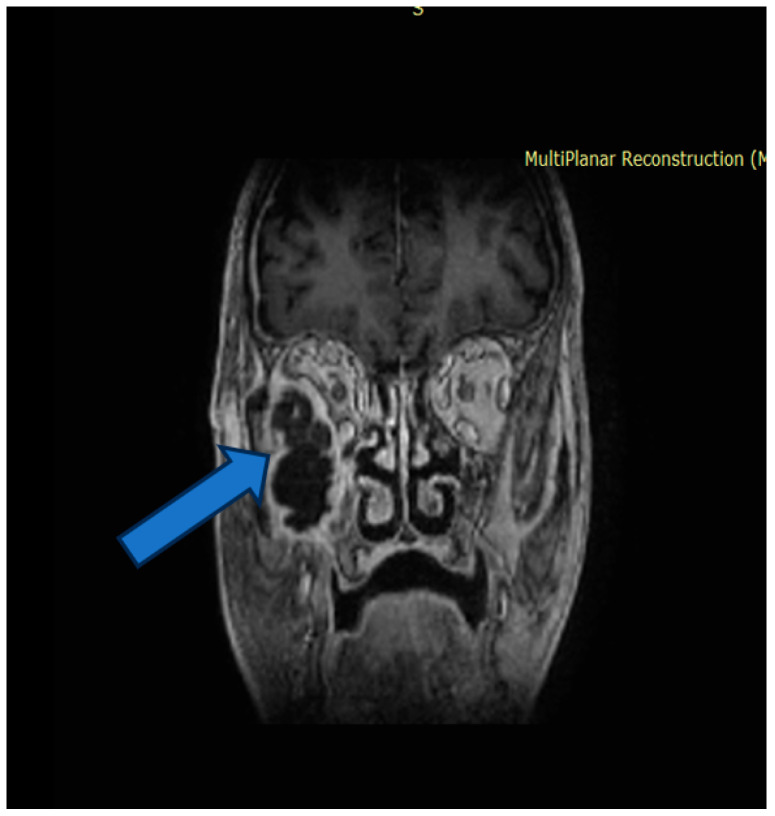
Magnetic resonance imaging before surgery. A mass-like structure with cystic and parenchymatous components. Invasion of the tumor in the right orbit (marked with arrow).

Due to the lack of clinical improvement, further imaging evaluation was performed. Magnetic resonance imaging (MRI) of the head revealed a heterogeneous mass with both cystic and solid components, including enhancing septa and peripheral gadolinium uptake. The lesion occupied the right maxillary sinus, with posterior extension towards the infratemporal fossa and superior invasion into the orbit, compressing orbital contents (Figure 1).

**Figure 2:**
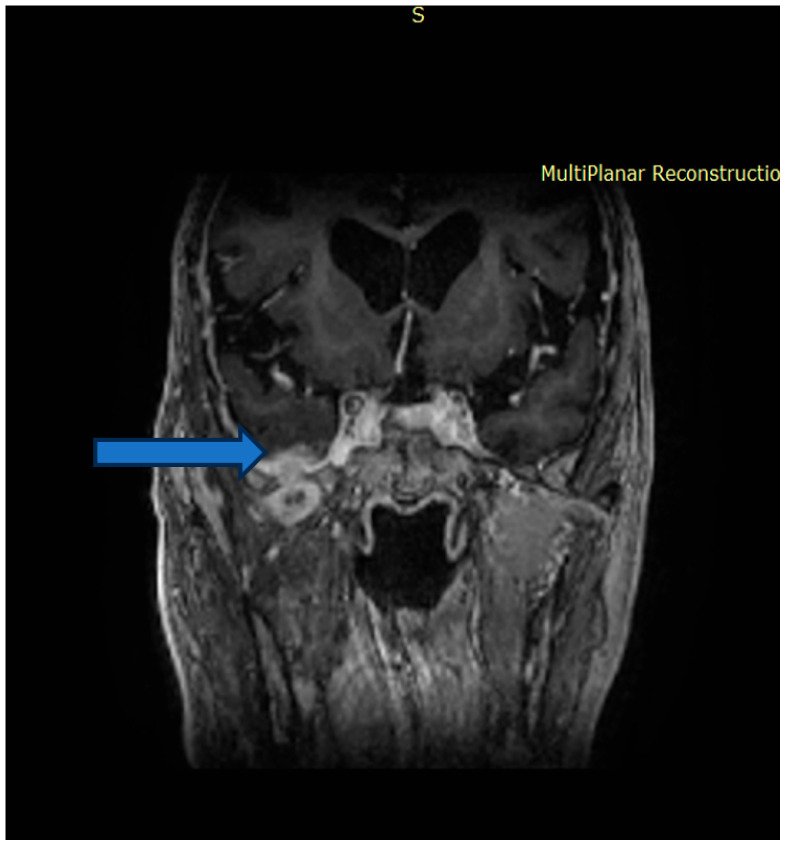
Magnetic resonance imaging before surgery. Invasion of the tumor in the dura mater (marked with arrow).

The tumor measured 45 × 46 × 34 mm (anteroposterior × transverse × craniocaudal). It infiltrated the lateral wall of the right maxillary sinus, the floor of the orbit, and the orbital surface of the sphenoid greater wing and showed clear dural invasion (Figure 2).

At this point, the patient and relatives were informed about the advanced stage and large volume of the tumor. Intracranial invasion of the tumor was also presented. The most critical argument against ablative surgery was the tumor’s invasion through the orbital surface of the ala major of the sphenoid bone and the dura mater. The second critical issue was the tumoral invasion of the orbit, which presumed orbital exenteration.

**Figure 3:**
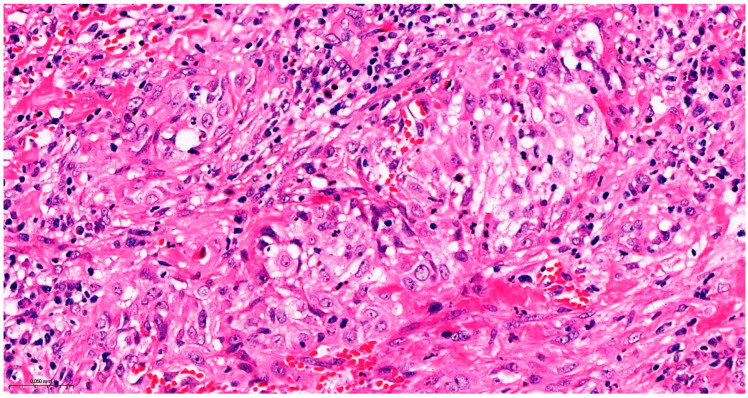
The invasive component consists of cells arranged in islands, with marked nuclear atypia and mitoses. Hematoxylin and Eosin, ×20.

**Figure 4:**
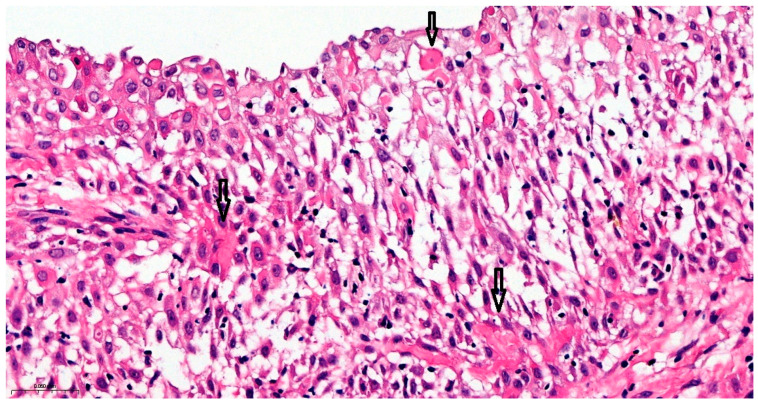
Atypical cells with clear cytoplasm and eosinophilic dentinoid material, marked with black arrows. Hematoxylin and Eosin, ×20.

**Figure 5:**
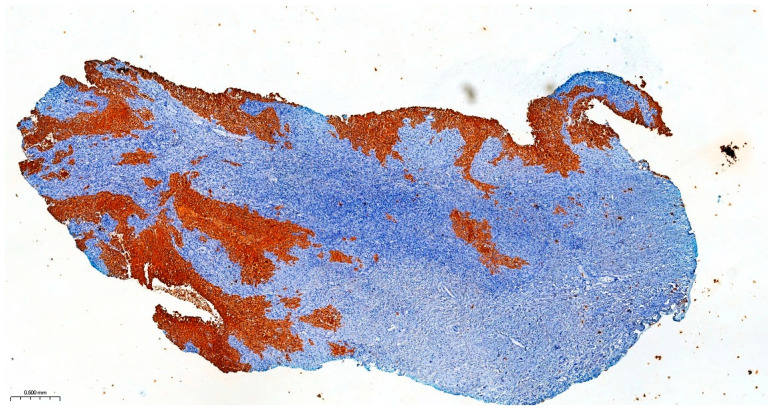
Immunohistochemical staining for keratin AE1/AE3 showing the cytoplasmic positivity of cells from the surface epithelium and the invasive component, ×2.

**Figure 6:**
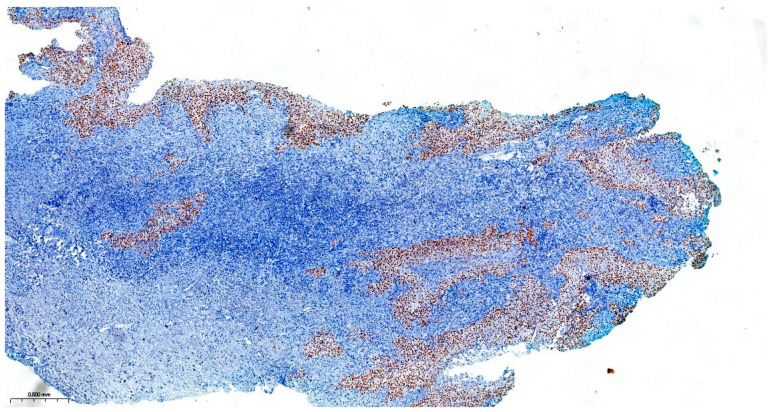
Immunohistochemical staining for p 63 showing the nuclear positivity of cells from the surface epithelium and the invasive component, ×2.

Precise diagnostic incisional biopsy was performed, reaching the tumor through a cutaneous approach at the infratemporal fossa. The biopsy showed the presence of an invasive carcinoma, arising from the epithelium of an odontogenic cyst. The epithelium of the cyst had the appearance of a non-keratinized squamous epithelium, stratified, similar to a stellate reticulum but without palisade at the periphery. Islands and isolated tumor cells were identified in the wall of the cystic formation, consisting of cells with a squamous appearance, including some with clear cytoplasm, nuclear atypia, and numerous mitoses. Small dentinoid material was identified between the atypical cells. Foci of necrosis and perineural invasion were identified (Figure 3 and Figure 4). Immunostaining demonstrated that the tumor cells were positive for keratin AE1/AE3, EMA, and p 63 but negative for vimentin. The final diagnosis was invasive carcinoma, with odontogenic characteristics and deposition of dentinoid-type material developed on the background of an odontogenic cyst (Figure 5 and Figure 6).

This preliminary histopathological diagnosis came as a surprise because based on the imagistic evaluation of the patient on which the borders of the tumor appeared well defined and partially cystic in structure, the operative team expected a benign tumor or, in the worst-case scenario, a border-like tumor. Based on these findings, ablative surgery raised a series of questions regarding the degree of infirmity after orbital exenteration, intra- and postoperative vital complications due to intracranial infiltration, postoperative evolution, and prognosis. The patient finally decided on ablative surgery in hopes of achieving pain reduction. From the patient’s point of view, vital intracranial complications, infirmity after orbital exenterations, and facial deformity were secondary issues. Given the complexity and extensive volume of the tumor, a multidisciplinary approach was required, involving oral and maxillofacial surgeons, a neurosurgeon, a pathologist, a psychologist, an ophthalmologist, and an anesthesiologist.

The potential risks and complications were thoroughly explained, and both the patient and his family confirmed their understanding and provided informed consent for the surgical intervention. The patient signed an informed consent form for surgery and general anesthesia.

### 2.1. First Step of Surgical Treatment

**Figure 7:**
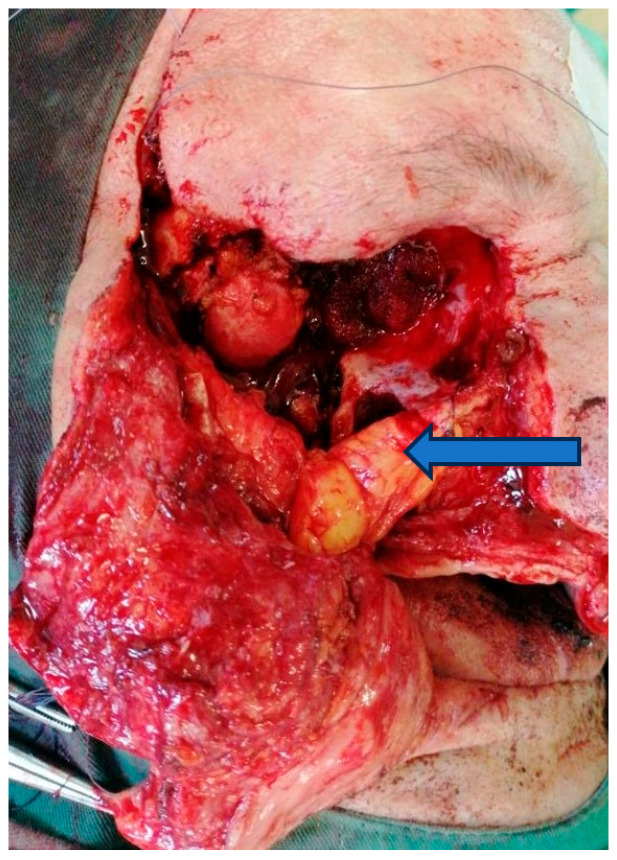
Surgical intervention. Facial defect after total maxillary resection and orbital exenteration and Bichat flap pad (marked with arrow).

General anesthesia with orotracheal intubation was provided. For better hemostasis control, the right external carotid artery was ligated. The tumor was accessed via a modified Weber–Ferguson incision placed on the right side of the face. After raising the cheek flap, maxillary bone was exposed. Total right maxillectomy, including zygomatic bone resection, was performed with orbital exenteration (Figure 7) and careful hemostasis. After orbital exenteration, it was possible to access the ala major of the sphenoid towards the orbital fasciae. At this stage, the neurosurgeon was able to resect the infiltrated bony surface along with the involved dura mater.

**Figure 8:**
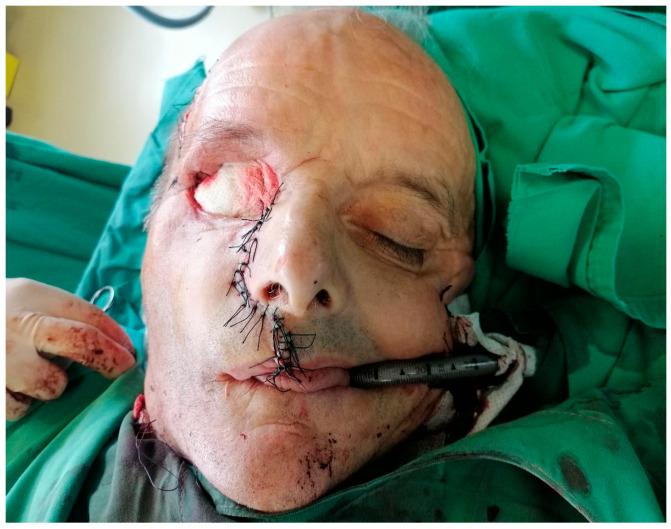
Postoperative view after resection.

To prevent cerebrospinal fluid leakage, the defect of the resected dura mater was protected with a collagen membrane (Lyoplant^®^, B. Braun, Melsungen, Germany). Towards the oral cavity, the large defect was closed with the Bichat flap pad. The cheek flap was placed on top of these and secured with non-resorbable sutures (Figure 8).

The cheek flap was repositioned, and gauze dressing was applied to achieve hemostasis and facilitate monitoring of local healing. All resection specimens, including bone, orbital tissues, and dura mater fragments, were immediately immersed in 10% neutral-buffered formalin and sent for histopathological examination.

The patient was monitored for 24 h in the intensive care unit, extubated the following day, and subsequently transferred to the Oral and Maxillofacial Surgery ward. The early postoperative course (first 3–4 days) was uneventful, with no signs of cerebrospinal fluid leakage, hemorrhage, or infection. Daily wound care, analgesic medication, and intravenous antibiotics were administered to prevent infection and ensure adequate postoperative comfort.

The patient remained hospitalized for a total of 14 days. Notably, the debilitating neuralgic pain resolved completely after surgery, and the patient reported significant improvement in comfort, psychological well-being, and quality of life and expressed satisfaction with the surgical outcome.

**Figure 9:**
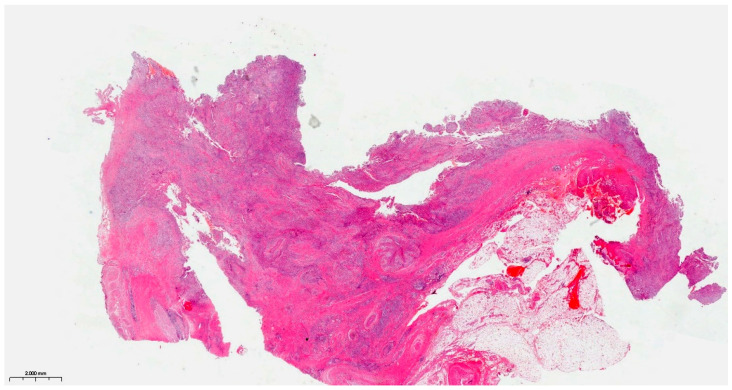
Section through the tumor fragment infiltrating the maxillary bone with complete destruction of the bone, Hematoxylin and Eosin, ×2.

**Figure 10:**
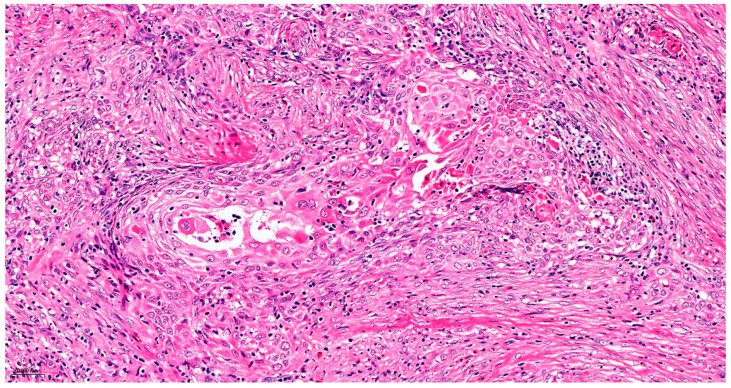
The invasive component with the aspect of squamous cell carcinoma, Hematoxylin and Eosin, ×20.

**Figure 11:**
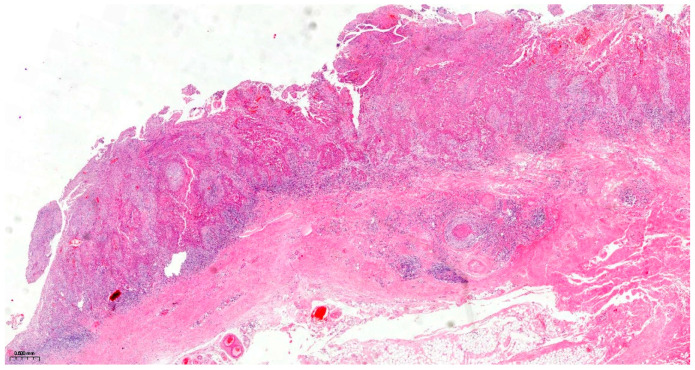
The cystic component of the tumor with squamous epithelium on the surface, Hematoxylin and Eosin, ×5.

**Figure 12:**
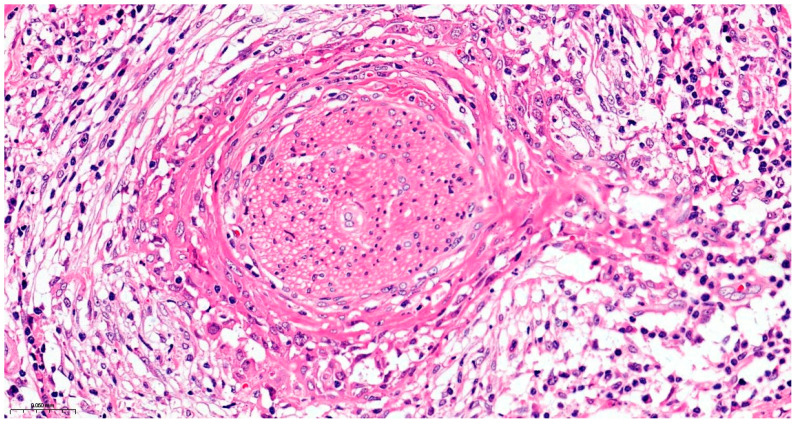
Perineural invasion, Hematoxylin and Eosin, ×10.

**Figure 13:**
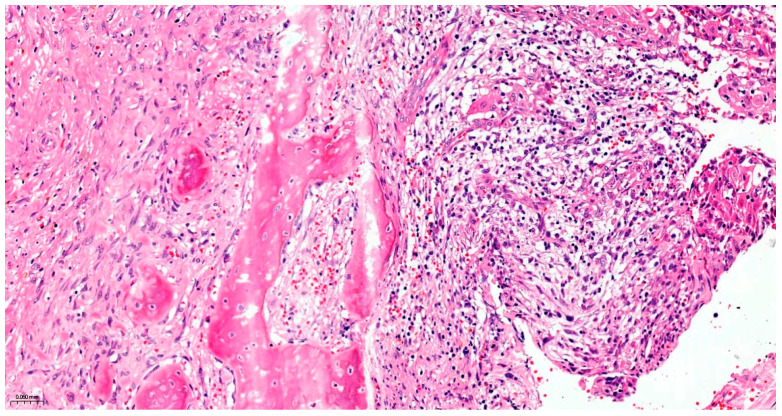
Invasion of the maxillary bone Hematoxylin and Eosin, ×10.

The resection surgical material sent for histopathological examination was fragmented, with some parts containing the maxillary bone structure and others containing the eyeball with eyelids and the optic nerve. The dura mater fragment was sent separately to ensure a deep margin of safety. All the examined fragments showed tumor invasion with a microscopic appearance similar to that described in the biopsy, including the cyst wall from which the tumor process started. Dentinoid material or ghost cells were not identified on any of the examined sections. No peripheral palisade was identified. The tumor destructively infiltrated the maxillary bone and the adipose tissue at the base of the orbit without directly invading the eyeball, eyelids, and optic nerve. The dura mater fragment was infiltrated along its entire length and thickness. Perineural invasions were identified, but no lymph vascular emboli were observed, and necrosis was present. As the piece was sent fragmented, the surgical resection margins could not be accurately evaluated. Due to the lack of a UICC/AJCC (Union for International Cancer Control/American Joint Committee on Cancer) staging system for malignant odontogenic tumors, a pTNM stage was not established, but the ICCR (International Collaboration on Cancer Reporting) reporting criteria for these tumors were followed. The final diagnosis was primary squamous intraosseous carcinoma, infiltrative in the maxillary bone, the base of the orbit, and dura mater, developed on the background of an odontogenic cyst, with necrosis and perineural invasions (Figure 9, Figure 10, Figure 11, Figure 12 and Figure 13).

### 2.2. Second Step of Surgical Treatment

**Figure 14:**
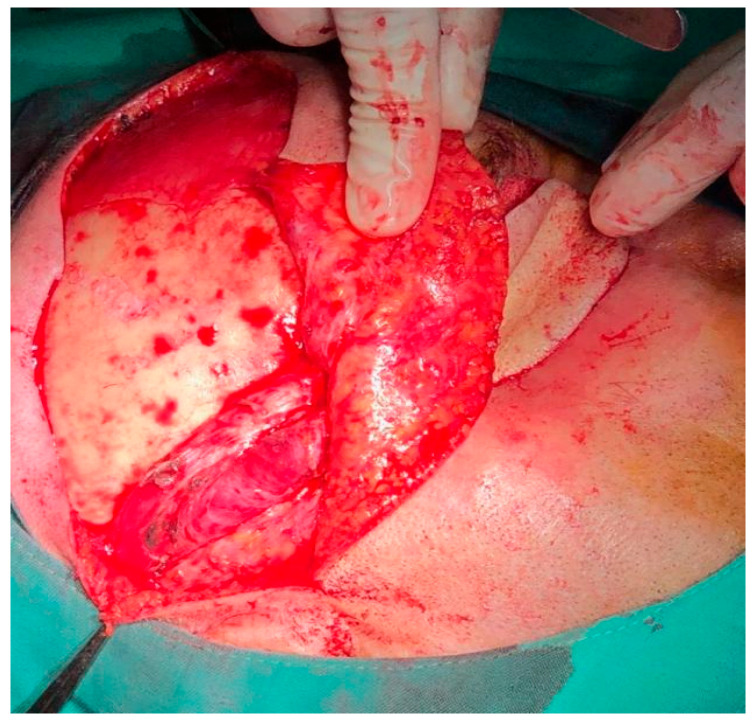
Second step surgery: intra operatory view.

**Figure 15:**
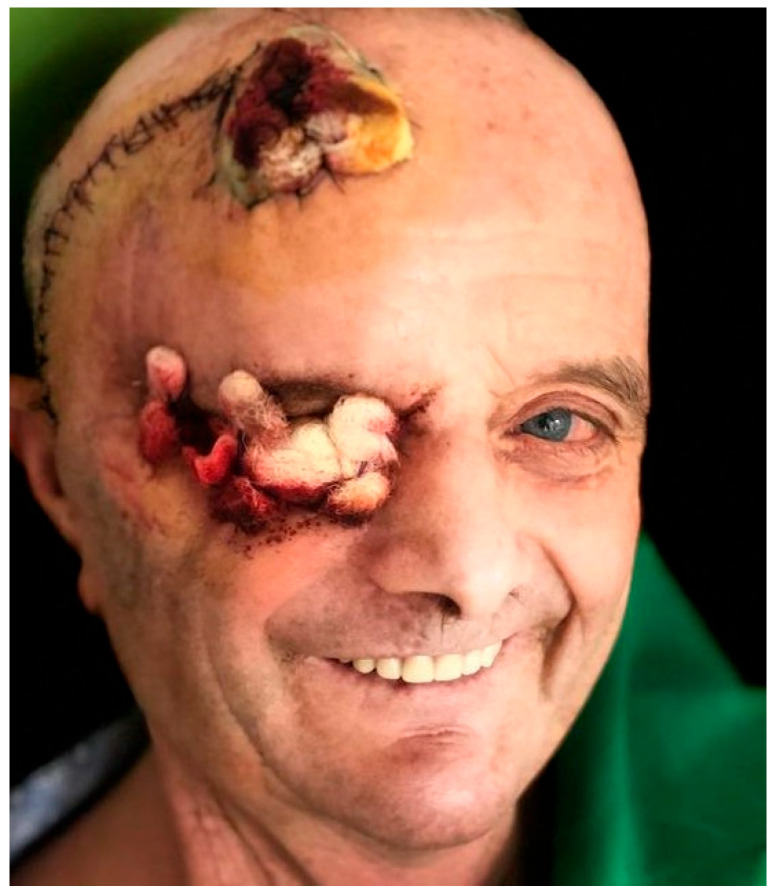
Postoperative view, second day after surgery, showing adequate protection for the dura and orbital cavity.

Six weeks after the initial tumor resection, the second stage of surgical management was performed to reconstruct the orbital cavity and protect the exposed cranial base. A temporoparietal fascial flap with a cutaneous island was harvested and rotated into the defect to provide vascularized soft tissue coverage (Figure 14). This reconstructive approach enhanced both the structural stability and the soft tissue quality in the region, offering adequate protection for the dura and orbital cavity (Figure 15).

The postoperative evolution was favorable, with satisfactory wound healing, no signs of infection or cerebrospinal fluid leakage, and complete resolution of neuropathic pain. The patient reported significant improvement in comfort, esthetics, and psychological well-being and was deemed fit to proceed with adjuvant oncologic treatment.

**Figure 16:**
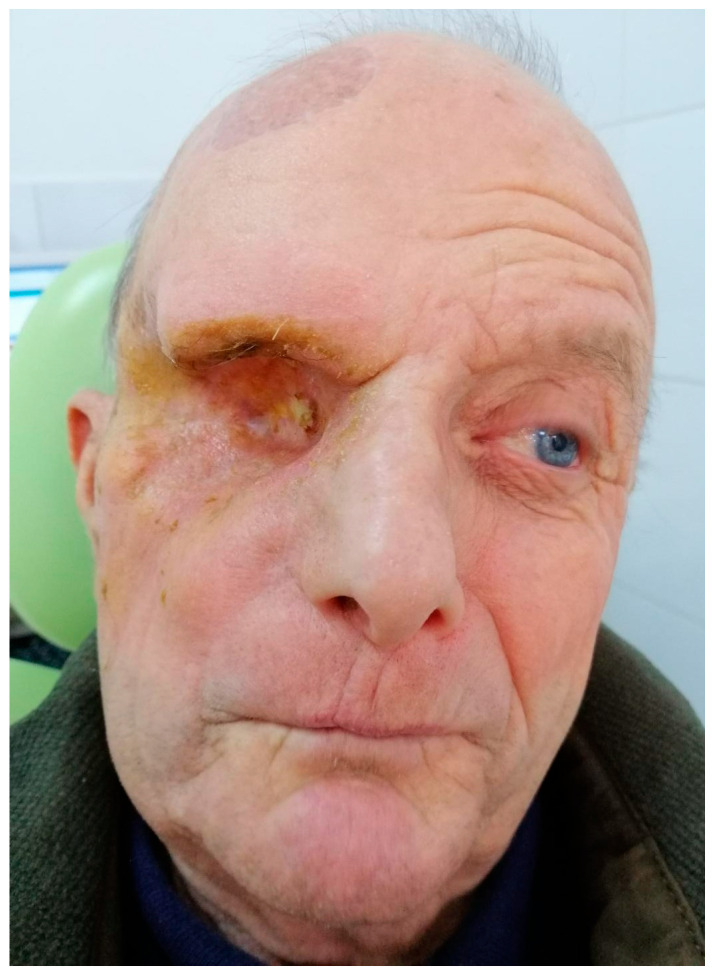
Thirtieth postoperative day after complete wound healing.

Fourteen days after the reconstructive procedure, the patient was referred to the oncology department. Intensity-Modulated Radiation Therapy with Volumetric Modulated Arc Therapy (IMRT/VMAT) was administered at a total dose of 66 Gy in 33 fractions, targeting the tumor bed and adjacent high-risk tissues. A one-month postoperative follow-up examination revealed stable reconstruction with satisfactory tissue integration, no wound complications, and good cosmetic outcomes (Figure 16).

### 2.3. Follow-Up

**Figure 17:**
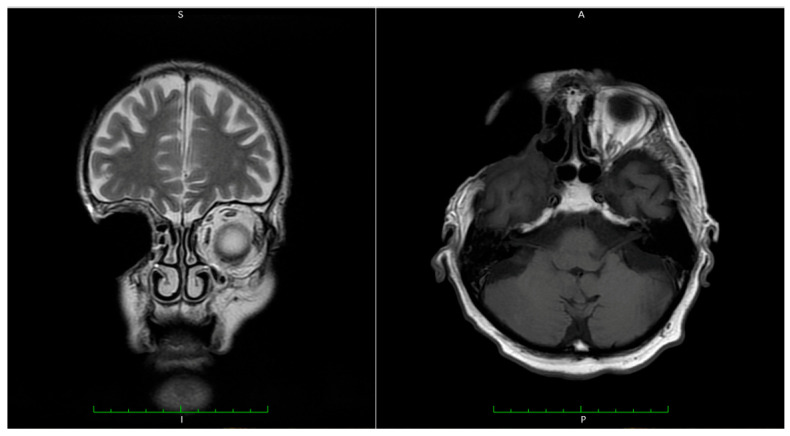
Magnetic resonance imaging (MRI) of the patient, 6 months postoperatively, with no local recurrence.

The patient underwent clinical examination every two months. Magnetic resonance imaging (MRI) revealed good healing of the dura mater and no local recurrence (Figure 17).

At 1.5 years postoperatively, the patient underwent positron emission tomography (PET), which showed no abnormal tracer uptake, indicating the absence of local or distant recurrence. Six months later (2.8 years after the initial diagnosis), we were informed by the patient’s family that he had passed away due to unrelated medical conditions, including acute abdomen and hepatobiliary complications.

In accordance with national legislation and at the request of the family, no post-mortem biopsy or autopsy was performed. As such, disease recurrence at the time of death could not be formally excluded; however, no clinical signs suggestive of tumor progression were observed during the previous follow-up consultations.

## 3. Discussion

Tumors with odontogenic differentiation are rare, and among them, carcinomas are exceptionally encountered in current practice. Because of this, there are difficulties in establishing diagnosis and in the therapeutic conduct.

The new 2024 WHO classification recognizes malignant and benign odontogenic tumors, including epithelial, mixed, epithelial and mesenchymal, and mesenchymal tumors. Sclerosing odontogenic carcinoma, ameloblastic carcinoma, clear cell and phantom cell odontogenic carcinoma, and NOS (not otherwise specified) primary intraosseous carcinoma are also recognized.

Despite the new WHO classification, there are still articles that use old terminology [8]. To avoid any inconvenience in understanding this specific and rarely met pathology and its behavior, we believe the primary definition of intraosseous carcinoma should be used.

Primary intraosseous carcinoma arises more commonly from preexisting odontogenic cysts or de novo from odontogenic rests within the jawbones. Two main differentiations can occur: the first type is a squamous cell carcinoma phenotype, and the other is an ameloblastomatous phenotype. In our case, the histopathological aspect of the tumor was of a squamous type, with some ameloblastic appearance, represented by clear cells and a follicular pattern but without any palisading of the peripheral cells or formation of stellate reticulum-like areas in the invasive component. Dentinoid-like material can be found in primary intraosseous carcinomas but is more abundant in clear cell odontogenic carcinoma, characterized by the presence of sheets and cords of epithelial cells with clear cytoplasm and dentinoid deposition. In our case, no dentinoid-like material was found in the resection specimen, representing another exceptional feature.

Unlike other malignant tumors, including those of the head and neck, there is no TNM staging system recommended by the UICC (Union for International Cancer Control)/AJCC (American Joint Committee on Cancer) for malignant odontogenic tumors. However, reporting according to the ICCR (International Collaboration on Cancer Reporting), which states that it is necessary to evaluate the tumor size during imaging analysis, is encouraged [9]. Some authors have proposed a staging system based on the size of the tumor, namely whether it is smaller or larger than 6 cm, together with the extension in the surrounding tissues (orbit or base of the skull), which seems to allow the prognostic stratification of patients with malignant odontogenic tumors [9].

The type of carcinoma is also reported according to the WHO classification, with the grading being recommended only for the intraosseous variant [10]. In addition to reporting vascular and perineural invasion, evaluating necrosis is also necessary, with its presence being considered a confirming element of malignancy [10]. In contrast to this, perineural invasion, although it must be reported, is not an element that can certify malignancy as it can also be found in other benign odontogenic tumors [10].

The status of surgical excision margins is a key element in reporting, with important prognostic implications [10]. Tumors with posterior extension have a reserved prognosis, especially if the surgical excision is incomplete at the level of the infratemporal fossa or the base of the skull. The massive extension of the tumor in the surrounding tissues, including towards the base of the skull, which also required the enucleation of the eyeball, did not allow the tumor to be excised in one piece, making the histopathological evaluation of the surgical excision margins impossible. The lack of correct information about the excision margins was compensated by additional radiotherapy [10].

In the case reported in this study, the lesion developed asymptomatically over a prolonged period. This was likely due to its location within the maxillary sinus, where the anatomical space allowed for silent progression. In certain cases of maxillary tumors, asymptomatic swelling of the vestibular region serves as the first warning sign, prompting radiologic investigation [11]. When tumors arise in the lower part of the maxillary sinus and are in proximity to the dental arch, symptoms such as tooth mobility or localized swelling may be easily mistaken for an infection. Incisions made for drainage in such cases are usually ineffective, underscoring the critical importance of imaging studies for establishing an accurate diagnosis [12].

Unnoticed and oligosymptomatic growth of benign lesions may lead to the development of malignant tumors, with their size making radical surgical treatment much more difficult, with a worsening prognosis. Their unnoticed growth and long-term oligosymptomatic nature are due to their localization and the characteristic pneumatization of the maxilla and intraosseous placement. Notably, the diagnosis of maxillary tumors is initially based on clinical symptomatology. Several cases in the literature with the absence of obvious clinical symptomatology, such as ulceration of the oral mucosa, face deformity, tooth loss, and bleeding, only present severe pain, making diagnosis more difficult as these cases are often confounded with trigeminal neuralgia [13]. In a recent study, Bashir, Atif et al. highlighted the most important clinical features of malignant tumor growth in the maxilla and concluded that these cases are insidious, with clinical signs presenting late in advanced tumor stages [14].

Liu et al. highlighted that when trigeminal neuralgia is suspected, detailed imaging should be performed along the nerve trajectory to determine whether malignant tumors are present in this area. The diagnosis is made when trigeminal neuralgia debuts with mild paresthesia, a common feature of malignant intraosseous tumor growth [15].

In the presented case, the tumor of the maxilla extended into the anterior cranial fossa, requiring a multidisciplinary team with mandatory participation of the neurosurgeon. As other authors have reported, treating such tumors is challenging [16,17] as these tumors were considered inoperable for many years. In our patient, the biggest challenge was relieving pain, but we achieved this after tumor resection.

For all patients with oral cancer, pain is the main complaint. Pain may become excruciating in advanced stages of cancer, which motivates patients to choose the most aggressive and challenging surgical treatments with a great amount of tissue loss and possible life-threatening complications [17]. Pain can also encourage patients to make higher-risk decisions [18,19]. Oral and facial defects after maxillectomy can be debilitating, causing difficulty in swallowing, feeding, and integration in social life. Nowadays, gold standard reconstruction involves free flap transfer, but obturator prosthesis can be an acceptable solution for elderly patients. In a similar case in which maxillectomy was performed, reconstruction was carried out with a local flap, namely the temporoparietal fascial flap, achieving good results [20,21].

Temporoparietal fascial flaps are frequently used for facial reconstruction because they are versatile and do not need further microsurgical skills, and they can also be used as skin island flaps. In this case, due to the boldness of the patient, there was no impairment due to hair loss, and the right orbit was reconstructed with optimal textured tissue [22].

PIOC in evolution destroys the bone and produces metastases in the vicinity of the tumor. Unlike the case presented by McDonald NA with metastases in the submandibular region, our case, located in the maxillary bone, was not associated with local or distant metastases [23]. Domenico Galetta reported a case of PIOC with distant metastases 7 years after mandibulectomy [24].

Due to the reduced case numbers and follow-ups of PIOC patients, there are still many uncertainties about the aggressiveness and recurrence potential of this tumor.

In particular, this case consisted of extension of the tumor to the cranial fossa and the relatively well-defined aspect of an impressive tumor, which evolved to such extent because of an odontogenic maxillary lesion. Finally, despite the risk, the patient wished for a radical approach due to his suffering. This case highlights the importance of including odontogenic malignancies in the differential diagnosis of atypical facial pain.

In the present case, the site of occurrence—within the maxillary sinus with extension to the orbital floor—lies outside the classic anatomic regions where odontogenic tumors are most commonly expected. However, the WHO (2024) [1] emphasizes that odontogenic epithelium may persist as developmental epithelial rests throughout the maxillary bone, including areas distant from the dental alveolus. These remnants can give rise to odontogenic cysts and, rarely, odontogenic carcinomas. Histopathology in our case demonstrated a cystic component lined by non-keratinized stratified squamous epithelium resembling odontogenic epithelium, together with an infiltrative squamous carcinoma showing focal dentinoid-type deposition—findings that cannot be explained by a sinus-derived carcinoma. The identification of these odontogenic features confirms that the tumor most likely originated from odontogenic rests entrapped within the maxilla, even though its expansion toward the maxillary sinus may give the impression of a sinus-based malignancy. This clarification is particularly important because maxillary PIOC is rare and may be misinterpreted as a primary maxillary sinus carcinoma unless its true odontogenic origin is emphasized.

## 4. Conclusions

PIOC of the maxillary bone remains a diagnostic and therapeutic challenge due to its rarity, heterogeneous clinical features, and variable biological behavior. Our maxillary case confirms that radical surgery with clear margins is the cornerstone of management, while the role of radiotherapy and chemotherapy remains uncertain. The present case emphasizes two key aspects: the risk of misdiagnosis as trigeminal neuralgia in early stages and the need for a multidisciplinary approach when the tumor extends to the orbit and cranial base. Clinicians should maintain a high index of suspicion in patients with atypical trigeminal pain and consider odontogenic malignancies in the differential diagnosis. Early imaging, timely surgical intervention, and individualized multidisciplinary care are crucial to improving patient outcomes and quality of life.

## Data Availability

Data supporting the findings of this review are available within the cited literature. Clinical details of the reported case are contained within the article.

## Abbreviations

The following abbreviations are used in this manuscript:

PIOC	Primary intraosseous carcinoma
MRI	Magnetic resonance imaging
UICC	Union for International Cancer Control
AJCC	American Joint Committee on Cancer
ICCR	International Collaboration on Cancer Reporting
